# A Predictive Model of Cognitive Function in Older Adults from a Native Amazonian Community: The Case of the Shipibo‐Konibo in Peru

**DOI:** 10.1002/alz70857_105263

**Published:** 2025-12-25

**Authors:** Claudia Cruzalegui‐Bazán, Patricio Castro‐Suarez, Martha Vilca‐Salas, Gerardo Luna‐Peralta, Sandra Uriol‐Alvino, Hamilet Huaccho‐Travezaño

**Affiliations:** ^1^ Sociedad Científica de San Fernando, Lima, Lima, Peru; ^2^ Universidad Nacional Mayor de San Marcos, Lima, Lima, Peru; ^3^ Universidad Peruana Cayetano Heredia, Lima, Lima, Peru

## Abstract

**Background:**

Cognitive decline, particularly in indigenous populations, is a significant concern for public health. The Shipibo‐Konibo community in the Peruvian Amazon faces challenges such as limited access to healthcare and education, which may impact cognitive health. This study aims to develop a predictive model for cognitive function in older adults from this community to inform preventive measures and interventions for Alzheimer's disease and other cognitive disorders.

**Method:**

A cross‐sectional study was conducted with 79 older adults (aged 60+) from the Shipibo‐Konibo community. Data collection was part of the CUMIS (Multidisciplinary University Research and Service Camp), organized by the Society of San Fernando and the Faculty of Medicine at the National University of San Marcos in December 2023. Cognitive function was assessed using the Mini‐Mental State Examination (MMSE). Diabetes and hypertension were self‐reported or confirmed through medical records, physical activity was measured using the International Physical Activity Questionnaire (IPAQ), depressive symptoms were assessed using the Geriatric Depression Scale (GDS), and nutritional status was evaluated using the Mini Nutritional Assessment (MNA). A linear regression model was used to develop a predictive model for cognitive performance, evaluating sociodemographic factors, lifestyle factors, medical history, and nutrition.

**Result:**

The predictive model revealed that age negatively impacted cognitive function (*p* = 0.047), with older participants showing lower MMSE scores and higher GDS stages. Education emerged as the strongest positive predictor (*p* < 0.001), indicating that higher education levels protect against cognitive decline. Additionally, physical activity significantly enhanced cognitive function (*p* = 0.0017), and nutritional status showed a positive relationship with cognitive health (*p* = 0.035). Chronic conditions such as diabetes, hypertension, and substance use (alcohol/tobacco) did not significantly impact cognitive performance. The model explained over 60% of the variance in cognitive function, providing a robust tool for predicting cognitive decline.

**Conclusion:**

The study emphasizes that age, education, physical activity, and nutritional status are critical predictors of cognitive function. These findings suggest that interventions promoting education, physical activity, and nutrition could mitigate cognitive decline in older adults, especially in underserved communities like the Shipibo‐Konibo. The model offers a valuable tool for personalized care and health interventions aimed at preventing Alzheimer's disease.

